# Antibody-Based Targeting of Cell Surface GRP94 Specifically Inhibits Cetuximab-Resistant Colorectal Cancer Growth

**DOI:** 10.3390/biom9110681

**Published:** 2019-11-01

**Authors:** Mee Hyun Jeoung, Taek-Keun Kim, Ji Woong Kim, Yea Bin Cho, Hee Jun Na, Byong Chul Yoo, Hyunbo Shim, Dong-Keun Song, Kyun Heo, Sukmook Lee

**Affiliations:** 1 Scripps Korea Antibody Institute, Chuncheon, Gangwon 24341, Korea; artist79@nate.com (M.H.J.); kimtk1981@gmail.com (T.-K.K.); hee7606@hauulbio.com (H.J.N.); 2 Biopharmaceutical Chemistry Major, School of Applied Chemistry, Kookmin University, Seoul 02707, Korea; jwk7853@gmail.com (J.W.K.); yjs03060@naver.com (Y.B.C.); kyunheo@kookmin.ac.kr (K.H.); 3 Research Institute, National Cancer Center, Goyang, Gyeonggi 10408, Korea; yoo_akh@ncc.re.kr; 4 Department of Bioinspired Science and Life Science, Ewha Womans University, Seoul 03760, Korea; hshim@ewha.ac.kr; 5 Department of Pharmacology, College of Medicine, Hallym University, Chuncheon, Gangwon 24252, Korea; dksong@hallym.ac.kr

**Keywords:** cetuximab resistance, GRP94, human antibody, colorectal cancer

## Abstract

Colorectal cancer (CRC) is one of the leading causes of cancer death worldwide. Cetuximab, a human/mouse chimeric monoclonal antibody, is effective in a limited number of CRC patients because of cetuximab resistance. This study aimed to identify novel therapeutic targets in cetuximab-resistant CRC in order to improve clinical outcomes. Through phage display technology, we isolated a fully human antibody strongly binding to the cetuximab-resistant HCT116 cell surface and identified the target antigen as glucose-regulated protein 94 (GRP94) using proteomic analysis. Short interfering RNA-mediated GRP94 knockdown showed that GRP94 plays a key role in HCT116 cell growth. In vitro functional studies revealed that the GRP94-blocking antibody we developed strongly inhibits the growth of various cetuximab-resistant CRC cell lines. We also demonstrated that GRP94 immunoglobulin G monotherapy significantly reduces HCT116 cell growth more potently compared to cetuximab, without severe toxicity in vivo. Therefore, cell surface GRP94 might be a potential novel therapeutic target in cetuximab-resistant CRC, and antibody-based targeting of GRP94 might be an effective strategy to suppress GRP94-expressing cetuximab-resistant CRC.

## 1. Introduction

Colorectal cancer (CRC) is the third most common cancer and the fourth leading cause of cancer-related deaths worldwide [1]. A number of chemotherapeutic agents, such as 5-fluorouracil (5-FU), irinotecan, and oxaliplatin, as well as their combinations, including the FOLFOX (leucovorin, 5-FU, and oxaliplatin) and FOLFIRI (leucovorin, 5-FU, and irinotecan) regimens, have been used as standard therapies for CRC [2,3]. However, despite their clinical efficacy, these chemotherapeutic agents inhibit DNA synthesis and/or disrupt microtubule structures, and are not targeted therapies. Thus, they result in widespread cytotoxicity and adverse effects, such as hair loss, diarrhea, low platelet counts, and sensory alterations [4].

Therapeutic antibody is the most effective targeted cancer therapy. Since the approval of mouse anti-CD3 monoclonal antibody (OKT3) by the Food and Drug Administration (FDA), the remarkable development of DNA recombinant technology has produced a variety of humanized and fully human antibodies. Recently, Imclone Systems Inc. first developed cetuximab, a recombinant mouse/human chimeric monoclonal antibody that targets the epidermal growth factor receptor (EGFR), to treat patients with EGFR- and wild-type ki-ras2 kirsten rat sarcoma viral oncogene homolog (KRAS)-expressing CRC [5]. However, despite being widely used in clinics, cetuximab is not effective as a single agent, and is recommended in combination with FOLFIRI or FOLFOX regimens. Heinemann et al. reported that FOLFIRI plus cetuximab could be the preferred first-line regimen for patients with metastatic colorectal cancer [6]. Furthermore, another unmet medical need of cetuximab cancer therapy is that it is only effective in a limited number of CRC patients. It is effective in approximately 10–20% of CRC patients; the other patients show cetuximab resistance due to gene mutations in downstream EGFR effectors, including KRAS, phosphoinositide-3-kinase catalytic subunit alpha (PI3KCA), phosphatase and tensin homolog (PTEN), and BRAF [7]. Cetuximab resistance caused by the gene mutations of several EGF downstream signaling molecules has been a major hurdle for treating CRC patients. Therefore, it is critical to identify novel potential therapeutic targets in CRC and develop novel therapeutics for improving the clinical outcomes of CRC therapy.

In this study, using phage display technology, we tried to isolate an antibody strongly binding to the surface of HCT116 cetuximab-resistant CRC from the human synthetic antibody library. Through proteomic analyses, we identified the target antigen as glucose-regulated protein 94 (GRP94). By overproducing and purifying a fully human monoclonal antibody specifically targeting GRP94, we demonstrated that the antibody targeting of cell surface GRP94 potently reduces the tumor growth of cetuximab-resistant CRC, without severe toxicity. Our findings suggested that an antibody-based modulation of the cell surface of GRP94 might be effective against GRP94-expressing CRC. Therefore, GRP94 might be a potential novel therapeutic target for antibody therapy.

## 2. Materials and Methods

### 2.1. Cell Culture

Human CRC cell lines, including HCT116, HT-29, LoVo, HCT-8, and Caco-2 were purchased from the Korean Cell Line Bank (Seoul, Korea). HCT116, HT-29, LoVo, and HCT-8 cells were maintained in Roswell Park Memorial Institute (RPMI) 1640 media (Gibco, Grand Island, NY, USA) supplemented with 10% (*v*/*v*) fetal bovine serum (Gibco) and 1% (*v*/*v*) penicillin/streptomycin (Gibco). Caco-2 cells were cultured in Dulbecco’s Modified Eagle Medium (DMEM) (Gibco) with the same supplements. Human umbilical vein endothelial cells (HUVECs; Lonza, Allendale, NJ, USA) were cultured in endothelial growth medium-2 (EGM-2; Lonza). All cells were maintained at 37 °C in a humidified incubator with 5% CO_2_ (Panasonic Healthcare Company, Wood Dale, IL, USA). Expi293 cells were cultured in Expi293 expression medium (Invitrogen, Carlsbad, CA, USA) in a humidified Multitron incubator shaker (Infors HT, Basel, Switzerland) at 37 °C with 8% CO_2_.

### 2.2. Selection of a High-Affinity Binder to HCT116 Cells Using Phage Display Technology

Human synthetic scFv library was reamplified [7]. Through four rounds of biopanning, a high-affinity binder to the surface of HCT116 cells was selected by phage enzyme immunoassays as previously described [8]. In order to generate immunoglobulin G (IgG) antibody, after the DNA sequencing of the scFv, each variable heavy and light chain gene of the selected scFv clone was cloned into the bicistronic mammalian expression vector pCDNA3.1 (Invitrogen). The IgG antibody (GRP94 IgG) was produced and purified as described previously [9].

### 2.3. Identification of a Target Antigen as GRP94

One milligram of total cell extract of HCT116 cells was applied to a HiTrap Q sepharose anion-exchange column. Unbound proteins (flow-through fractions) were collected, and bound proteins were then eluted at a flow rate of 0.5 mL/min using an increasing gradient of NaCl (0-1 M). The fractions were separated by sodium dodecyl sulfate-polyacrylamide gel electrophoresis (SDS-PAGE) and then subjected to Coomassie Brilliant Blue staining or immunoblot analysis with 20 µg/mL of the prepared IgG, respectively. The protein band corresponding to the band detected in immunoblot analysis was excised from the gel and digested with trypsin (Roche) for 6 h at 37 °C. The masses of tryptic peptides were determined by matrix-assisted laser desorption/ionization-time of flight (MALDI-TOF) mass spectrometry with better than 50 ppm mass accuracy on an average. Using the amino acid sequences and the mass numbers of the tryptic peptides, the SWISS-PROT database was searched for a protein match.

### 2.4. Immunoblot Analysis

Twenty microliters of each elute or 15 µg of cell lysates were resolved by SDS-PAGE and transferred onto nitrocellulose membranes using a wet transfer system (GE Healthcare Life Sciences, Piscataway, NJ, USA) GRP94 or β-actin (loading control) was detected by incubation with 20 µg/mL GRP94 IgG, anti-GRP94 antibody (1:200; Santa Cruz Biotechnology, Dallas, TX, USA), or anti-β-actin monoclonal antibody (1:3000; Santa Cruz Biotechnology) and then with horseradish peroxidase (HRP)-conjugated anti-human lambda light chain antibody (1:1000; Bethyl Laboratories, Montgomery, TX) or goat anti-mouse or rabbit secondary antibody (1:5000; Santa Cruz Biotechnology). Protein bands were visualized using SuperSignal West Pico chemiluminescent substrate (Pierce, Rockford, IL, USA) according to the manufacturer’s instructions.

### 2.5. Enzyme-Linked Immunosorbent Assay (ELISA)

Each well of a 96-well plate was coated with 0.1 μg of recombinant human GRP94 (rhGRP94) (Sino Biological Inc., Beijing, China), blocked with 3% (*w*/*v*) bovine serum albumin (BSA) in phosphate buffered saline (PBS) for 1 h at 37 °C, and incubated with 20 μg/mL GRP94 IgG for 2 h at room temperature. Plates were washed with PBS thrice, and 100 µL of 3,3′,5,5′-tetramethylbenzidine (TMB) substrate solution was added to each well. Optical densities were measured at 450 nm using a VICTOR X4 microplate reader (PerkinElmer, Waltham, MA, USA).

### 2.6. Transfection

HCT116 cells plated with a density of 2 × 10^5^ cells/well in six-well plates were transiently transfected with ON-TARGET plus Smart pool short interfering RNA (siRNA) specific to human GRP94 (Thermo Fisher Scientific, Lafayette, CO, USA) using Lipofectamine RNAiMAX transfection reagent (Thermo Fisher Scientific) according to the manufacturer’s instructions. For antibody overproduction, Expi293 cells were transiently transfected with an expression vector encoding GRP94 antibody using ExpiFectamine (Invitrogen) according to the manufacturer’s instructions.

### 2.7. Flow Cytometry

To evaluate GRP94 IgG binding to CRC cells, 2 × 10^5^ HCT116, HT29, Lovo, HCT-8, and Caco-2 CRC cells were fixed with 4% (*w*/*v*) paraformaldehyde (PFA), blocked with PBS containing 1% (*w*/*v*) BSA, and stained with 20 µg/mL GRP94 IgG for 1 h. Cells were incubated with Alexa Fluor 488-labeled anti-human Fab antibody (1:1000; Invitrogen) for 1 h. The effects of GRP94 IgG on endothelial cell activation were evaluated by incubating 2 × 10^5^ HUVECs in the presence or absence of 20 ng/mL human tumor necrosis factor-α (Millipore) or 20 μg/mL GRP94 IgG for 24 h. After blocking with PBS containing 1% (*w*/*v*) BSA for 1 h at room temperature, cells were incubated with 2 μg per well of anti-intercellular cell adhesion molecule-1 (ICAM-1) or anti-vascular cell adhesion molecule-1 (VCAM-1) antibody for 1 h at 37 °C and then with Alexa Fluor 488-conjugated anti-rabbit IgG (1:1000; Invitrogen) for 1 h at 37 °C. Samples were analyzed by flow cytometry (FACSCalibur, BD Bioscience). Data were analyzed using FlowJo software (TreeStar, Ashland, OR, USA).

### 2.8. Immunocytochemistry

HCT116 cells (density 5 × 10^4^) that were grown on 0.1% (*w*/*v*) gelatin-coated glass coverslips (Marienfeld-Superior, Lauda-Königshofen, Germany) were fixed with 4% (*w*/*v*) PFA, blocked with PBS containing 3% (*w*/*v*) BSA for 1 h, and incubated in the presence or absence of 20 μg/mL GRP94 IgG or anti-GRP94 polyclonal antibody (1:50; Santa Cruz Biotechnology) for 2 h at 37 °C. HUVECs (density 5 × 10^4^) that were grown on 0.1% (*w*/*v*) gelatin-coated glass coverslips (Marienfeld-Superior, Paul Marienfeld GmbH & Co. KG, Lauda-Königshofen, Germany) were incubated in the presence or absence of 20 μg/mL GRP94 IgG for 24 h at 37 °C. The cells were fixed in 4% (*w*/*v*) PFA, blocked with PBS containing 5% (*w*/*v*) BSA and 0.1% (*v*/*v*) Triton X-100 for 1 h at 37 °C, and then incubated with one unit/well rhodamine–phalloidin (Molecular Probes) and 0.1 μg/mL Hoechst 33,258 (Sigma-Aldrich, St Louis, MO, USA) for 1 h at room temperature. Images were acquired with a FluoView FV300 confocal microscope (Olympus, Cypress, CA, USA).

### 2.9. Real-Time Measurement of Antibody and Antigen Interactions

Biolayer interferometry (BLI) assays were performed using an Octet^®^ RED96 system (ForteBio/Pall Life Sciences, Menlo Park, CA, USA) as described previously [10,11]. Cells were incubated with fluorescein isothiocyanate (FITC)-labeled anti-human Fab (1:1000; Sigma-Aldrich, St Louis, MO, USA) or Alex 546-labeled anti-rabbit antibody (1:1000; Cell Signaling Technology) for 1 h at room temperature. Images were acquired using a FluoView FV300 confocal microscope (Olympus, Tokyo, Japan).

### 2.10. In Vitro Measurement of CRC Cell Growth

To examine the effect of GRP94 knockdown on HCT116 cell growth, 5 × 10^3^ HCT116 cells that were transfected with scrambled siRNA or with GRP94 siRNA were seeded into wells of 96-well plates. To examine the effect of GRP94 IgG on CRC cell growth in vitro, 5 × 10^3^ HCT116, HT29, LoVo, HCT-8, or Caco-2 CRC cells were seeded into wells of 96-well plates in the presence or absence of 100 μg/mL cetuximab or GRP94 IgG. Cell growth was measured for 60 h using an IncuCyte FLR live content imaging system (Essen Bioscience, Ann Arbor, MI, USA).

### 2.11. HUVEC Viability Assays

HUVECs (density 5 × 10^3^) were placed in 0.1% (*w*/*v*) gelatin-coated wells of a 96-well plate and incubated in EGM-2 in the presence or absence of 20 μg/mL GRP94 IgG or 36 μg/mL 5-fluorouracil for 48 h at 37 °C. Cell viability was determined using the Cell Counting Kit-8 (Dojindo Laboratories, Rockville, MD, USA) according to the manufacturer’s instructions. The final optical density was measured at 450 nm using a spectrophotometer (VICTOR X4, PerkinElmer, Norwalk, CT, USA).

### 2.12. In Vivo Mouse Experiments

All experiments were performed following protocols approved by IACUC and in accordance with the guidelines of Hallym University (Hallym 2017-72). To evaluate the effect of GRP94 IgG on HCT116 tumor growth, BALB/c-nude mice were subcutaneously injected with 3 × 10^6^ HCT116 cells. After 7 days, 10 mg/kg GRP94 IgG (*n* = 7), cetuximab (*n* = 7), or vehicle (*n* = 10) was intravenously injected twice weekly. Mice were weighed and tumor sizes were measured once per week up to day 42.

### 2.13. In Vivo Toxicity Testing

In vivo toxicity testing was performed as described previously [12]. Bagg albino (BALB)/c-nude mice (*n* = 4) were injected intravenously twice weekly with or without 10 mg/kg GRP94 IgG, and their body weights were measured weekly. After 42 days, mice were sacrificed and blood samples were collected. Serum glutamate oxaloacetate transaminase (GOT), glutamate pyruvate transaminase (GPT), total bilirubin (TBIL), blood urea nitrogen (BUN), and creatinine (CRE) were measured using a Fuji Dri-Chem 3500 biochemistry analyzer (Fujifilm, Tokyo, Japan).

### 2.14. Statistical Analysis

Data were analyzed using two-tailed Student’s t-tests for comparison between two groups and one-way analysis of variance with Bonferroni correction for multiple comparisons in GraphPad Prism 5.0 (GraphPad Software, La Jolla, CA, USA). Data are presented as mean ± SEM. *p*-values < 0.05 were considered statistically significant.

## 3. Results

### 3.1. Selection of a GRP94-Specific Fully Human Monoclonal Antibody

**Figure 1:**
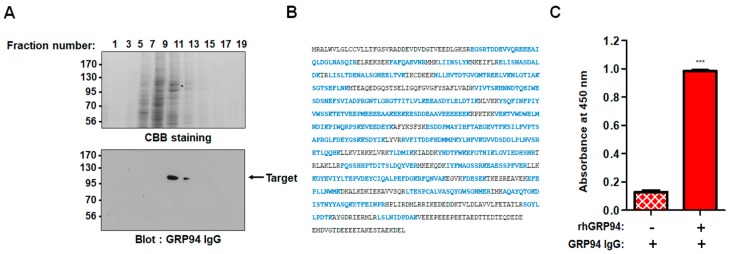
A 100-kDa protein recognized by glucose-regulated protein 94 (GRP94) immunoglobin G (IgG) was identified as human GRP94. (**A**) HCT116 total cell extract was subjected to ion-exchange column chromatography using a HiTrap Q sepharose column. (Upper panel) The fractionated proteins were visualized by Coomassie Brilliant Blue staining. (Lower panel) An immunoblot showing that the enriched 100-kDa protein was recognized by GRP94 IgG. The 100-kDa protein band (*) from the Coomassie-stained gel was excised and trypsinized. The resulting tryptic peptides were subjected to MALDI-TOF mass spectrometry. (**B**) Amino acid sequences in the 100-kDa protein that are identical to human GRP94 sequences are highlighted in blue. (**C**) The wells of a 96-well microtiter plate were coated with rhGRP94, and the binding of GRP94 IgG to rhGRP94 was evaluated by ELISA. Values represent mean ± SEM of triplicate measurements from one of three independent experiments. *** *p* < 0.001.

Using phage display technology, we isolated a single-chain fragment variable (scFv) clone strongly bound to the surface of HCT116 cells, a cetuximab-resistant CRC cell line, from the human synthetic scFv library. After conversion of the selected scFv clone to immunoglobulin G (IgG) form, we identified the target antigen. To do so, the total cell extract of HCT116 cells was fractionated by ion-exchange column chromatography with a HiTrap Q sepharose column, and the fractions were subjected to immunoblot analysis. The selected IgG antibody specifically recognized a protein with a relative molecular mass of ~100 kDa (Figure 1A). The protein band was excised from the gel, trypsinized, and subjected to MALDI-TOF mass spectrometry. The molecular masses of the peptides derived from the 100-kDa protein were almost identical to the calculated masses of theoretically predicted tryptic peptides of human GRP94. Further, the analyzed peptides covered ~65% of the human GRP94 protein sequence (Figure 1B). To further confirm the identity of the antigen detected by the selected IgG antibody, with commercially available recombinant human GRP94 (rhGRP94), we then performed ELISA (Figure 1C). We found that the IgG antibody specifically and strongly recognizes rhGRP94. These results showed that the high-affinity HCT116 cell surface binder we isolated is a GRP94-specific fully human monoclonal IgG antibody (GRP94 IgG).

### 3.2. Characterization of GRP94 IgG

**Figure 2:**
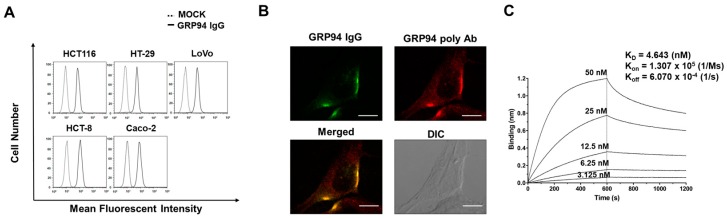
Characterization of GRP94 IgG. (**A**) Binding of GRP94 IgG to colorectal cancer (CRC) cells was verified by flow cytometry in the absence (MOCK, dashed line) or presence (solid line) of GRP94 IgG. (**B**) Immunocytochemical staining of HCT116 cells with GRP94 IgG or GRP94 polyclonal antibody (GRP94 poly ab). GRP94 IgG localization was examined using confocal microscopy. Scale bar = 20 µm. (**C**) The binding affinity of GRP94 IgG binding to rhGRP94 was measured by biolayer interferometry (BLI) using the Octet RED96 system.

To investigate the binding of GRP94 IgG to endogenous GRP94 on CRC cells, we performed flow cytometry and found that GRP94 IgG binds strongly to endogenous GRP94 on the surfaces of five CRC cell lines, including HCT116, HT29, LoVo, HCT-8, and Caco-2 cells (Figure 2A). To further confirm the specific binding of GRP94 IgG to endogenous GRP94 on CRC cells, we performed immunocytochemistry with GRP94 IgG and a commercially available GRP94 polyclonal antibody (positive control) on HCT116 cells. Similar staining patterns were observed with both antibodies (Figure 2B), further indicating that GRP94 IgG binds specifically to endogenous GRP94. Then, using biolayer interferometry, we demonstrated that the GRP94 IgG binds specifically to rhGRP94 with a dissociation constant (Kd) of ~4.6 nM (Figure 2C).

### 3.3. Role of GRP94 in Cetuximab-Resistant CRC Cell Growth

**Figure 3:**
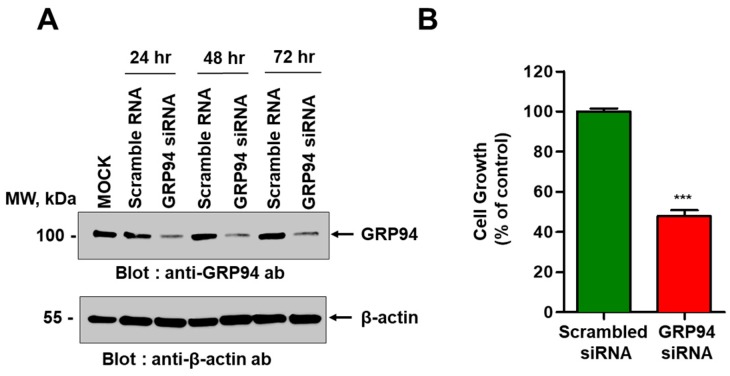
Effect of short interfering RNA (siRNA)-mediated GRP94 knockdown on HCT116 cell growth. (**A**) Immunoblot analysis showing GRP94 expression in HCT116 cells transfected with scrambled siRNA or GRP94-siRNA. (**B**) Growth of HCT116 cells transfected with scrambled siRNA or GRP94-siRNA was quantified and expressed as a percentage of the control value. Data are presented as mean ± SEM of triplicate measurements from one of three independent experiments. *** *p* < 0.001.

To examine the role of GRP94 in cetuximab-resistant CRC cell growth, we performed a short interfering RNA (siRNA)-mediated knockdown of GRP94 in HCT116 cells. First, we confirmed the reduced GRP94 expression in HCT116 cells using immunoblot analysis (Figure 3A). We measured the effect of GRP94 knockdown on HCT116 cell growth. GRP94 knockdown significantly decreased HCT116 cell growth (Figure 3B), indicating that GRP94 plays an important role in HCT116 cell growth. These results suggest that GRP94 is a key player in cetuximab-resistant CRC cell growth.

### 3.4. Effect of GRP94 IgG on Cetuximab-Resistant CRC Cell Growth

**Figure 4:**
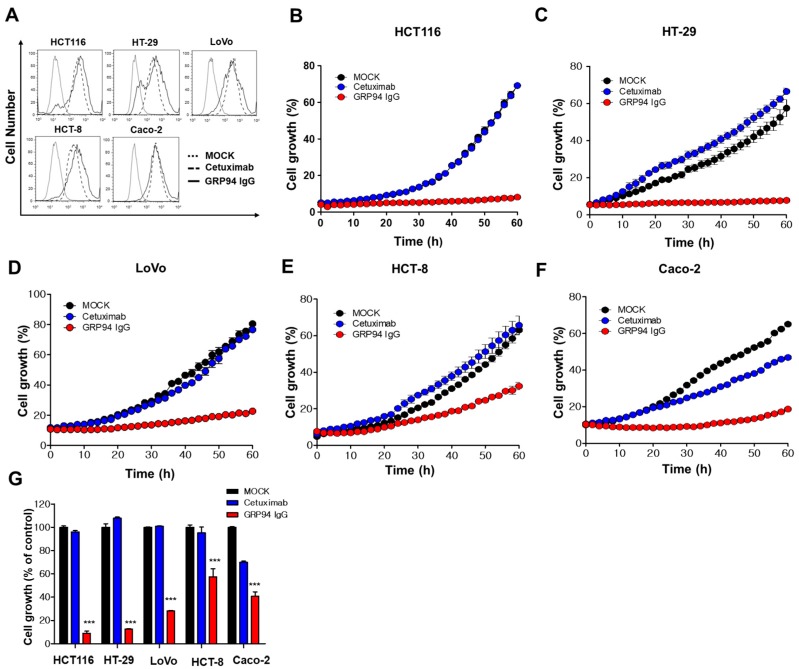
Effect of GRP94 IgG on CRC cell growth. (**A**) HCT116, HT-29, LoVo, HCT-8, and Caco-2 cells were subjected to flow cytometry in the absence (dotted line) or presence (dashed line) of cetuximab or GRP94 IgG (solid line). (**B**) HCT116, (**C**) HT-29, (**D**) LoVo, (**E**) HCT-8, and (**F**) Caco-2 CRC cells were seeded into a 96-well microtiter plate and incubated in the absence (MOCK) or presence of GRP94 IgG or cetuximab, and then cell growth was measured using the Incucyte FLR live content imaging system. (**G**) Cell growth was expressed as a percentage of the control (MOCK) cell growth. Values represent mean ± SEM of triplicate measurements from one of three independent experiments. *** *p* < 0.001.

To determine the effect of GRP94 IgG on cetuximab-resistant CRC cell growth, we used flow cytometry to determine the binding extent of GRP94 IgG or cetuximab on five CRC cell lines cetuximab-resistant HCT116, HT-29, LoVo, and HCT-8 cells and cetuximab-sensitive Caco-2 cells. GRP94 IgG strongly bound to the surface of all CRC cell lines, similar to cetuximab (Figure 4A). We also evaluated the inhibitory effect of GRP94 IgG or cetuximab on CRC cell growth by culturing the cells in the presence or absence of GRP94 IgG or cetuximab. CRC cell growth was monitored in real time live-cell imaging. GRP94 IgG significantly and more potently inhibited the growth of cetuximab-resistant HCT116, HT-29, LoVo, and HCT-8 cell lines compared to cetuximab, which had no effect or a weak effect (Figure 4B–E). In addition, in cetuximab-sensitive Caco-2 cells, both GRP94 IgG and cetuximab inhibited cell growth (Figure 4F). These results showed that GRP94 has a significant inhibitory effect on CRC cell growth (Figure 4G), suggesting that GRP94 IgG might be a potent inhibitor of cetuximab-resistant CRC growth in vivo.

### 3.5. Endothelial Cell Toxicity of GRP94 IgG

**Figure 5:**
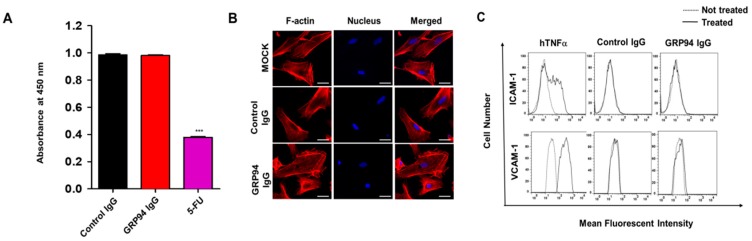
Effect of GRP94 IgG on endothelial cell toxicity. (**A**) Human umbilical vein endothelial cells (HUVECs) were incubated in the presence of control IgG, GRP94 IgG, or 5-fluorouracil (5-FU) for 2 days. Cell viability was evaluated by measuring absorbance at 450 nm. Values represent mean ± SEM of triplicate measurements from one of three independent experiments. *** *p* < 0.001. (**B**) HUVECs cultured in the presence or absence of GRP94 IgG were stained with rhodamine–phalloidin and Hoechst and then examined by confocal microscopy. Scale bar = 25 µm. (**C**) After HUVECs were cultured in the presence (solid line) or absence (dashed line) of human tumor necrosis factor-alpha (hTNEα), control IgG, or GRP94 IgG, they were stained with anti-intercellular cell adhesion molecule-1 (ICAM-1) (upper) or anti-vascular cell adhesion molecule-1 (VCAM-1) (lower) polyclonal antibodies and analyzed by flow cytometry. Results are representative of two independent experiments.

To evaluate the endothelial cell cytotoxicity of GRP94 IgG, we first evaluated the viability of HUVECs in the presence or absence of GRP94 IgG, control IgG (negative control), and 5-FU (positive control). GRP94 IgG was not cytotoxic to HUVECs, while 5-FU significantly reduced HUVEC viability (Figure 5A). We monitored morphological changes in HUVECs in the presence or absence of GRP94 IgG by labeling the actin cytoskeleton with rhodamine–phalloidin, which is a fluorescent dye that stains F-actin, and found that GRP94 IgG does not induce significant morphological changes in HUVECs (Figure 5B). To investigate the effect of GRP94 IgG on endothelial cell activation, which is an initial inflammatory response to harmful stimuli, we treated HUVECs with GRP94 IgG or control IgG and monitored HUVEC activation by measuring expression of the endothelial cell activation markers vascular cell adhesion molecule-1 (VCAM-1) and intercellular cell adhesion molecule-1 (ICAM-1). We used human tumor necrosis factor-alpha (hTNFα) as a positive control for endothelial cell activation. GRP94 IgG did not significantly affect HUVEC activation, while hTNFα induced HUVEC activation (Figure 5C). These results suggested that GRP94 IgG might not induce endothelial cell toxicity in vivo.

### 3.6. In Vivo Efficacy and Toxicity of GRP94 IgG

**Figure 6:**
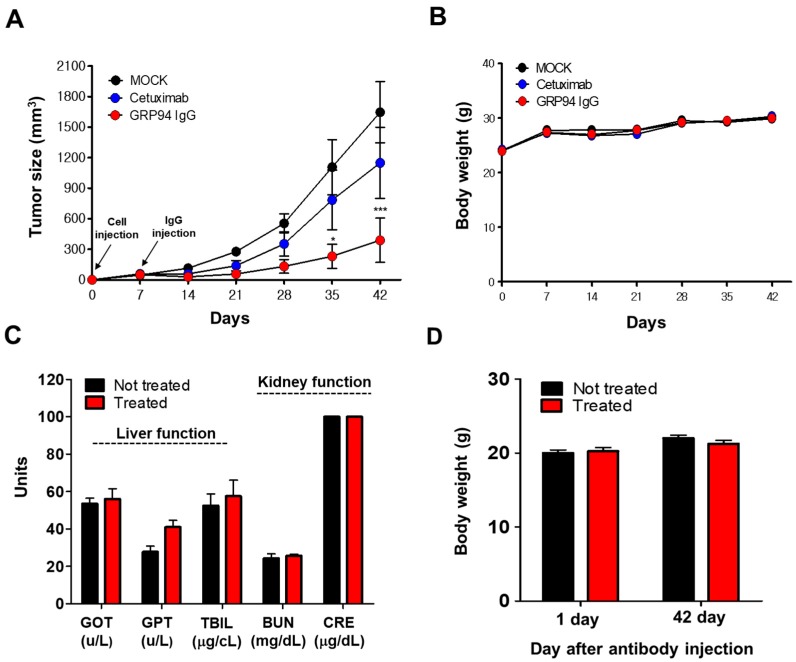
Evaluation of in vivo efficacy and toxicity of GRP94 IgG. (**A**) GRP94 IgG or cetuximab was injected intravenously into HCT116-xenografted mice twice weekly. Tumor sizes in the untreated group (MOCK) and in groups treated with the antibody were monitored until day 42. Values represent mean ± SEM of duplicate measurements from one of two independent experiments. * *p* < 0.05, *** *p* < 0.001. (**B**) Total body weights of mice in the MOCK group and in groups treated with GRP94 IgG or cetuximab were measured twice weekly. (**C**) In vivo toxicity was measured by changes in GOT, GPT, TBIL, BUN, and CRE concentrations measured 42 days after antibody injection. (**D**) In vivo toxicity can also be detected by changes in the body weights of mice from days 1–42 after antibody injection.

To investigate the effect of GRP94 IgG on cetuximab-resistant CRC cell growth, we generated an HCT116 xenograft mouse model by subcutaneously injecting HCT116 cells into nude mice. Then, GRP94 IgG or cetuximab was injected intravenously twice weekly for 7–42 days after HCT116 cell injection. Tumor sizes and body weights were monitored during this period. GRP94 IgG significantly suppressed HCT116 cell growth more effectively than cetuximab (Figure 6A). In addition, GRP94 IgG did not affect the total body weights of the mice, indicating that GRP94 IgG is not significantly toxic to mice (Figure 6B). To evaluate the in vivo toxicity of GRP94 IgG, we injected control IgG or GRP94 IgG intravenously twice weekly into normal mice and then monitored their liver and kidney functions and body weights. Liver function was monitored by measuring serum concentrations of GOT, GPT, and TBIL. Kidney function was monitored by measuring BUN and CRE concentrations. No significant changes in liver function, kidney function (Figure 6C), or body weight (Figure 6D) were observed. These results suggested that GRP94 IgG might be effective in suppressing cetuximab-resistant CRC cell growth, without severe in vivo toxicity.

## 4. Discussion

The identification of novel potential therapeutic targets in cetuximab-resistant CRC is important for improving clinical outcomes for CRC patients. In this study, using phage display technology and proteomic analysis for the first time, we isolated a human antibody strongly binding to the surface of HCT116 cells, a cetuximab-resistant CRC cell line, and identified the target antigen as human GRP94. Efficacy and toxicity evaluation studies demonstrated that this antibody can effectively suppress not only the growth of a variety of cetuximab-resistant CRC cells in vitro, but also HCT116 cell growth in vivo without severe toxicity. Therefore, on the basis of our findings, we believe that GRP94 might be a novel potential therapeutic target in cetuximab-resistant CRC and that antibody-based targeting of GRP94 might be effective in reducing CRC growth in cetuximab resistance cases.

In this study, GRP94 IgG, a GRP94-specific fully human monoclonal antibody that we developed, seemed highly specific to GRP94. Proteomic analysis identified the 100-kDa protein recognized by GRP94 IgG as human GRP94. ELISA and BLI results revealed that GRP94 IgG specifically binds to purified rhGRP94 with nanomolar affinity. Further, immunocytochemistry data showing a similar staining pattern of both GRP94 IgG and commercially available anti-GRP94 polyclonal antibody in HCT116 cells also supported our hypothesis.

GRP94 is a heat shock protein 90 (HSP90)-like protein and is also known as gp96, endoplasmin, Tra-1, or HSP90B1. Traditionally, GRP94 is a molecular chaperone that stabilizes newly synthesized or misfolded proteins inside cells. It is a stress-inducible protein and is primarily expressed in the endoplasmic reticulum (ER) lumen in response to ER stress [13,14,15,16]. Although some studies have reported that GRP94 exists on the membrane surfaces of several types of cells, the role of cell surface GRP94 in tumor malignancy has not yet been clearly defined. In this study, we proposed that cell surface GRP94 might be a key regulator in tumor growth. Several lines of evidence support our hypothesis. Li et al. reported that a monoclonal antibody selectively targeting GRP94 also shows antiproliferative effects on human EGFR 2 (HER2)-expressing breast cancer cell growth, both in vitro and in vivo [17]. In this study, we developed a GRP94-specific human monoclonal antibody strongly binding to the membrane surface of HCT116 cells. Flow cytometry analysis revealed that GRP94 IgG can specifically recognize GRP94 binding to the surface of various cetuximab-resistant CRC cells, such as HT-29, LoVo, HCT-8, and HCT116 cells. siRNA-mediated GRP94 knockdown demonstrated that GRP94 is a key regulator of HCT116 CRC cell growth. In addition, the antibody targeting of membrane GRP94 specifically suppressed not only the in vitro growth of HCT116, HT-29, LoVo, and HCT-8 cells, but also the in vivo growth of HCT116 cells. Studies have reported that GRP94 specifically forms a complex with several membrane proteins, including integrin subunits, HER2, and low-density lipoprotein receptor-related protein 6 (LRP6) [17,18,19]. These findings led us to speculate that the cell surface of GRP94 might interact with membrane proteins on cancer cells and further stimulate mitogenic signals in tumor malignancy.

GRP94 is an adenosine triphosphate (ATP)-dependent molecular chaperones comprising an N-terminal ATPase domain, a middle domain, and a C-terminal dimerization domain. Due to the close relationship of GRP94 with tumor malignancy, so far, many pan-specific HSP90 and GRP94-selecive small chemical inhibitors specifically inhibiting ATP binding have been developed [20]. However, despite their superior efficacy, they could induce toxicities and off-target effects. In this regard, it is believed that antibody therapy can be an alternative strategy for suppressing GRP94-mediated tumor growth.

EGFR is a key therapeutic target of antibody therapy for CRC [21]. Studies have reported that EGFR is overexpressed in CRC, and that its overexpression correlates with poor prognosis for CRC patients [22]. EGF signaling plays a pivotal role in tumor growth and progression in CRC [23]. When EGF binds to EGFR, homodimerization or heterodimerization of the receptor, in turn, leads to the activation of two main downstream signaling pathways, RAS-RAF-mitogen-activated protein kinase (RAS-RAF-MAP) and phosphatidylinositol 3-kinase–phosphatase and tensin homolog–protein kinase B (PI3K-PTEN-Akt), for regulating gene expression in CRC cells [24]. Furthermore, despite its limited efficacy in CRC treatment, cetuximab has been widely used to treat patients with EGFR- and wild-type KRAS-expressing CRC in clinics. In this study, we also suggested that cell surface GRP94 might be a novel potential therapeutic target in cetuximab-resistant CRC and that antibody targeting of GRP94 might be an effective strategy for suppressing tumor growth in cetuximab-resistant CRC. Through the generation of a human monoclonal antibody specifically targeting cell surface GRP94, antibody-based modulation of the cell surface of GRP94 inhibits the growth of cetuximab-resistant HCT116, HT-29, LoVo, and HCT-8 cells, and further suppresses HCT116 cell growth in a xenograft animal model. Moreover, we elucidated that GRP94 IgG has no effects on HUVEC viability, HUVEC morphological changes, and HUVEC activation, showing no severe endothelial cell toxicity. Additionally, GRP94 IgG does not induce significant changes in liver or kidney function or body weight in mouse models. Although further studies are required, our finding that GRP94 IgG has better efficacy in inhibiting the growth of cetuximab-sensitive Caco-2 cells compared to cetuximab also shows the possibility that the antibody targeting of cell surface GRP94 might simultaneously inhibit CRC cell growth in both cetuximab responders and cetuximab nonresponders. These results provide several clues for understanding the potential application of antibody-based targeting of GRP94 to treat CRC patients.

In summary, cell surface GRP94 is a potential novel therapeutic target in CRC, and antibody targeting of GRP94 might be an effective strategy for suppressing tumor growth in GRP94-mediated CRC. On the basis of currently available evidence, GRP94 IgG likely binds to GRP94 overexpressed on the CRC cell surface within a tumor microenvironment. GRP94 IgG specifically and effectively blocks CRC cell growth without severe toxicity. In future studies, we will investigate the mechanism of action of GRP94 IgG in more detail, and evaluate the in vivo efficacy of GRP94 IgG alone and in combination with other chemotherapeutic agents against GRP94-expressing CRC.

## 5. Conclusions

The cell surface of GRP94 is critical for regulating cetuximab-resistant CRC cell growth. Further, the antibody targeting of cell surface GRP94 might be an alternative strategy to overcome cetuximab resistance for antibody therapy. This study was the first study to identify the cell surface of GRP94 as a novel potential therapeutic target in cetuximab-resistant CRC, and also to show that the antibody-based modulation of GRP94 might be, at least in part, helpful in overcoming cetuximab resistance.

## 6. Patents

There is no patent resulting from the work reported in this manuscript.
